# The role of natural exosomes from SHED-MSC in immunoregulation of M0/M1 polarized macrophage cells

**DOI:** 10.3389/fimmu.2025.1550280

**Published:** 2025-02-07

**Authors:** Ali Fallah, Abasalt Hosseinzadeh Colagar, Ayyoob Khosravi, Azadeh Mohammad-Hasani, Mohsen Saeidi

**Affiliations:** ^1^ Department of Molecular and Cell Biology, Faculty of Basic Science, University of Mazandaran, Babolsar, Iran; ^2^ Stem Cell Research Centre, Golestan University of Medical Sciences, Gorgan, Iran; ^3^ Department of Molecular Medicine, Faculty of Advanced Medical Technologies, Golestan University of Medical Sciences, Gorgan, Iran; ^4^ Department of Immunology, School of Medicine, Golestan University of Medical Sciences, Gorgan, Iran

**Keywords:** SHED-MSCs, exosome, immunoregulatory macromolecules, macrophage polarization, immunomodulation

## Abstract

**Introduction:**

Exosomes (EXOs) as a targeted cell-free therapy could offer a new therapeutic strategy for immune-mediated inflammatory diseases, due to their stability and ease of storage and handling. This study focused on exosomes derived from stem cells of human exfoliated deciduous teeth (SHED-MSC-EXOs) and their role in managing the balance of immunoregulatory macromolecules that play a role in the underlying immunoregulatory mechanisms in THP-1-derived M0/M1 macrophage cells.

**Methods:**

Flow cytometry confirmed the expression of CD14, CD68, CD80, and CD86 markers in these macrophages. Following morphological and survival assessments, culture supernatants from SHED-MSCs were used to isolate exosomes. Once the exosomes were verified, Calcein AM-labeled EXOs were introduced to the macrophage cells. The immunoregulatory macromolecules were assessed by analyzing surface markers, cytokine production, and pro- and antioxidant activity.

**Results:**

Macrophages treated with exosomes exhibited immunomodulatory effects akin to those treated with dexamethasone. The levels of anti-inflammatory and antioxidant markers, including CD206, Arg-1, IL-10, TGF-β, TAC, CAT, and SOD, which act as immunosuppressive macromolecules, were elevated. In contrast, there was a reduction in pro-inflammatory and pro-oxidant markers, including CD80, CD81, IL-6R, IL-12, TNF-α, MDA, and NO, which act as immunostimulatory macromolecules (P < 0.05).

**Discussion:**

The findings suggest that exosomes derived from SHED-MSC can skew M0/M1 macrophages to the M2 phenotype and inhibit M1 polarization. These nanovesicles, with their distinct physical properties and ability to penetrate target cells, may prove beneficial in conditions involving the depletion of M2 macrophages and M1 macrophage-induced diseases, potentially aiding in the reduction of inflammation and tissue injury.

## Graphical Abstract

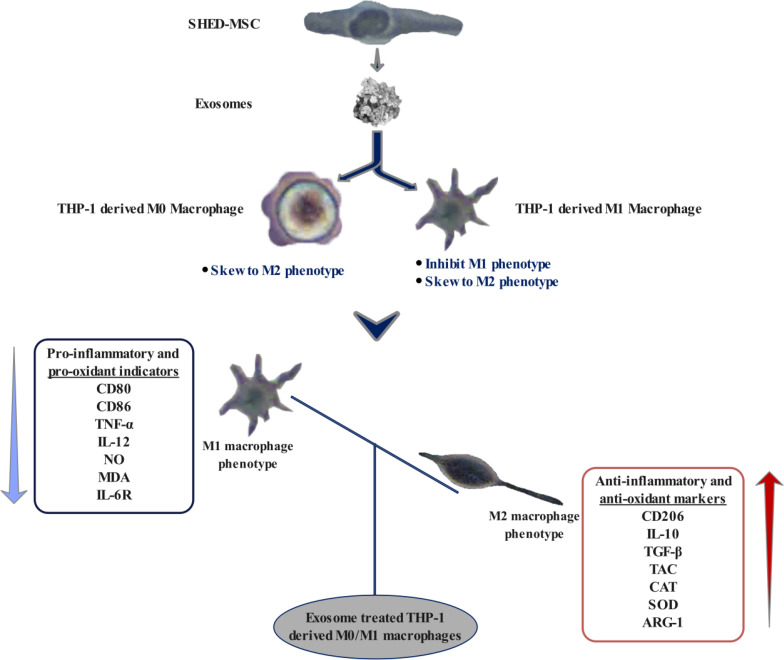

## Highlights

- Exosomes derived from SHED-MSC decrease the levels of immunostimulatory macromolecules in M0/M1 polarized macrophages.
- The exosomes released by SHED-MSC enhance the presence of immunosuppressive macromolecules in THP-1 cell-derived macrophages.
- SHED-MSC-natural exosomes mimic the immunomodulatory effects of dexamethasone on human macrophages.

## 1 Introduction

Macrophages’ adaptability allows them to alter their characteristics in reaction to various environmental stimuli and cytokines. The M0 and M1 macrophage polarization switching by mesenchymal stem cells (MSCs) serve as a method to inhibit the immune response (1, 2). The interaction between mesenchymal stem cells and pro-inflammatory macrophage cells (M0/M1) can be categorized as either direct (Cell to cell) or indirect (Paracrine secretion) contact (3). Studies indicate the immunoregulatory capability of MSCs relies significantly on their paracrine secretion (4). Growth factors, cytokines, chemokines, extracellular matrix components, and metabolic products have been identified as functional molecules of MSCs in different therapeutic contexts. These functional molecules can be released as soluble factors and extracellular vesicles.

Exosomes, a subtype of extracellular vesicles, could mimic the effects of parental cells and play a significant role in the paracrine mechanism (5, 6). Exosomes are naturally occurring nano-sized particles that have gained attention as potential cell-free therapeutic agents for autoimmune and inflammatory diseases due to their low immunogenicity and beneficial physical attributes such as their nano-scale size and lipid membrane (7, 8). EXOs are crucial in cell-cell communication in various physiological and pathological processes (9). Exosomes play a crucial role in regulating immune functions, particularly in macrophages, and they affect the polarization of these cells. Exosomes from various cell types, such as MSCs and tumor cells, can direct macrophages toward either pro-inflammatory (M1) or anti-inflammatory (M2) states (10, 11). Exosomes from MSCs promote M2 polarization, which supports tissue repair and reduces inflammation (12). Indeed, exosomes contain bioactive molecules like proteins, lipids, and RNA (including microRNAs) that macrophages can take up, leading to alterations in gene expression and modulation of the immune response (13). Certain microRNAs found in exosomes can inhibit pro-inflammatory signaling in macrophages, reducing inflammatory cytokine production (14). Exosomes can also induce a tolerant state in macrophages, making them less reactive to future stimuli and reducing the possibility of excessive immune responses (11). In some cases, exosomes can enhance the phagocytic activity of macrophages, improving their capacity to clear pathogens and debris (15).

An imbalance in the polarization of macrophages, specifically between M1 and M2 types, is often linked to the onset of autoimmune diseases, as macrophages are crucial in the inflammatory response (16). Therefore, targeting macrophage polarization with MSC-Exos could be a promising therapeutic approach. Our previous computational analysis showed that Exosomal microRNA obtained from stem cells derived from human-exfoliated deciduous teeth (SHED-MSC-EXO-miRNAs) impact the inflammatory mediators, the balance of oxidative stress, and the macrophage polarization (17).

This research explores the crucial function of natural exosomes derived from SHED-MSC as a cell-free treatment for regulating the levels of immunoregulatory macromolecules in M0/M1 polarized macrophages. To clarify the immunoregulatory signaling pathways, the THP-1 cell model was used to produce M0 and M1 macrophages. The immunomodulatory process was investigated using an *in vitro* approach, utilizing dexamethasone (Dex) as a reference point (positive control) to suppress M1 and promote M2 polarization.

## 2 Methods

### 2.1 Study design

**Figure 1:**
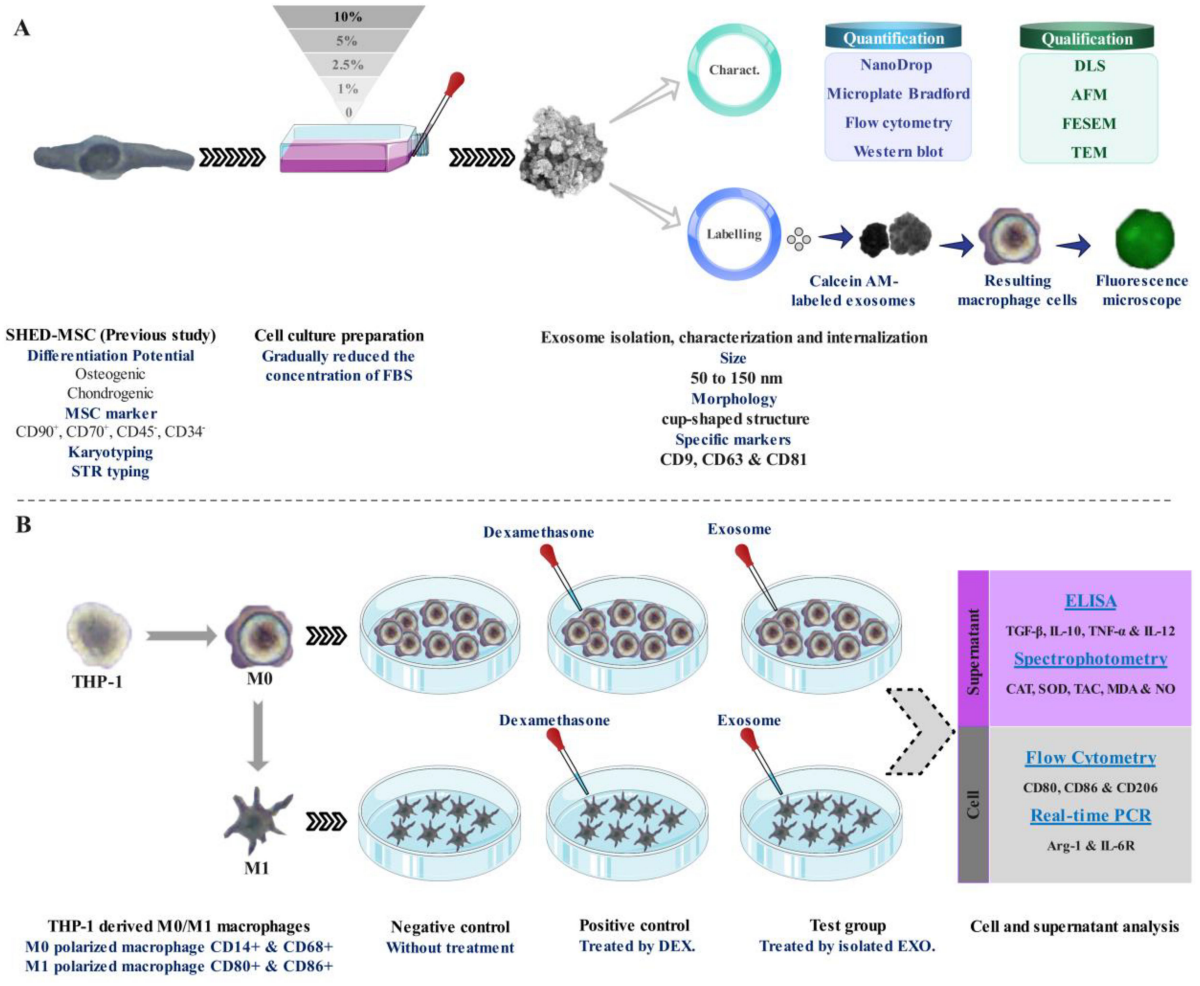
Schematic representation of the research. **(A)** Isolation, confirmation, and internalization of SHED-MSC-derived exosomes. **(B)** The use of SHED-MSC-EXOs follows the differentiation/polarization of THP-1 into macrophages M0/M1.

As illustrated in **Figure 1**, Following morphological and survival investigations and exosome isolation from the culture supernatants of SHED-MSCs, these nano-scale extracellular vesicles were confirmed by using various methods, including the Nanodrop spectrophotometer, micro-Bradford assay, Dynamic Light Scattering (DLS), Field Emission Scanning Electron Microscopy (FESEM), Transmission Electron Microscopy (TEM), Flow cytometry, and Western blotting (**Figure 1A**). After the internalization of EXOs using a derivative of the fluorescent molecule calcein called Calcein AM, the effects of these vesicles on THP-1 derived M0/M1 macrophages were examined through several methods, including Flow cytometry, Enzyme-linked immunosorbent assay (ELISA), Real-Time PCR, and Spectrophotometry (**Figure 1B**). The equilibrium between immunostimulatory and immunosuppressive macromolecules, which function as immunoregulatory macromolecules, was assessed by examining the expression of surface molecules, the release of cytokines, and the levels of pro- and antioxidant markers. Therefore, in every group (negative control, positive control, and Test) the levels of pro-inflammatory and pro-oxidant markers (such as CD80, CD86, IL-12, TNF-α, MDA, NO, and IL-6R) and the presence of anti-inflammatory and antioxidant markers (including CD206, IL-10, TGF-β, TAC, CAT, SOD, and ARG-1) were assessed.

### 2.2 Cell line culture

The SHED-MSCs were extracted from the healthy third molars of a 12-year-old girl, with written consent, at the School of Dentistry, Golestan University of Medical Sciences. The procedure for isolating human dental pulp stem cells (hDPSCs) was performed according to the approved protocol by Dr. Khosravi at the Faculty of New Technologies (18). After being transferred from the Pasteur Institute of Iran, the human monocyte cell line THP-1 and SHED-MSC were cultured in DMEM F12 medium with 10% FBS (Gibco Co.) and 1% antibiotics (100 U/mL penicillin G; 0.1 mg/mL streptomycin, Sigma-Aldrich Co.). The cells were used during passages 6 to 7 and kept in a humid state at 37°C with 5% CO_2_, with the culture medium being replaced every two days.

### 2.3 Exosome isolation

To adapt SHED-MSCs to a serum-free environment, we gradually reduced the concentration of FBS in the culture medium until it was eliminated. Once the cells reached 50% confluency, we decreased FBS levels from 10% to 5%, then to 2.5%, followed by 1%, and finally to eliminated FBS. These steps were carried out every 48 hours. The supernatant was collected After incubating the cells in serum-free media for 48 hours. SHED-MSC-EXOs were isolated using an Exocib exosome isolation kit (Cibbiotech, Tehran, Iran) by the manufacturer’s guidelines. In brief, we collected the cell culture supernatant and centrifuged it at 3,000 rpm at 4°C for 10 minutes to remove dead cells and debris. Then, we added Exocib solution to the supernatant and stored the mixture overnight at 4°C. Afterward, we centrifuged it at 3,000 rpm for 40 minutes at 4°C. To store the EXO pellets, 200 μL of 10 mM PBS pH 7 was added and stored at approximately -70°C or utilized for additional analysis.

### 2.4 The quantification of isolated exosomes

A nanodrop spectrophotometer (Thermo Fisher Scientific Inc., USA) and micro-Bradford assay were used to quantify the purified exosomes. To demonstrate protein concentration with nanodrop, approximately 2 µL of EXO solvent was used as a reference, followed by sample measurement at 280 nm. The results were then presented graphically. The micro-Bradford method employed the manufacturer’s instructions, using a BSA standard curve (Bio-Rad Co. CA). The absorbance was assessed at 595 nm using a spectrophotometer.

### 2.5 Exosome size analysis and visualization using DLS, AFM, FESEM, and TEM

The purified EXOs were diluted at a ratio of 1:100 in de-ionized water and exhibited no evidence of lysis or degradation, indicating their stability. The EXOs’ size distribution was evaluated using DLS Zetasizer (Malvern, Worcestershire, UK). The AFM visualization of the separated EXOs was achieved by dissolving them 1:100 in deionized water and then spread on freshly cleaved mica sheets. After that, SHED-MSC-EXOs were washed with de-ionized water and dried using a mild stream of Nitrogen. silicon probes were used for amplitude-modulated (AM–AFM) and phase-modulated (PM–AFM) tapping modes (MPP-13220-W, Spring Constant, k~200 Nm-1; Veeco). The isolated EXOs were transferred on silicon wafers, the wafer edges were ground with silver paint and cleaned with UV light. Then, these particles were coated with iridium at 20 mA for approximately 15 s to improve visibility in high-resolution FESEM (Magellan™400L, FEI Co. USA) using low-latency energy (1.5 kV @ 3.1 pA). The isolated exosomes suspension (5 μL) was transferred to a piece of parafilm for TEM imaging. Subsequently, a formvar-coated copper grid was gently placed on the droplet for 20 minutes at room temperature. The copper grid was quickly blotted with filter paper and then transferred to 4% paraformaldehyde (PFA) in 0.1 M sodium phosphate buffer, pH 7.3. The grid was then washed for one minute in 10 mM PBS, pH 7.4, and then placed in a solution of 1% glutaraldehyde in 0.1 M sodium phosphate buffer for 5 minutes. The grid was immersed in distilled water for 2 minutes to remove excess liquid. Before being placed in 1% uranyl acetate for 20 s, the grid was washed in 10 mM PBS, pH 7.4. Finally, any excess uranyl acetate was removed by blotting, and Images of grids were captured using a JEM-1400Plus Transmission Electron Microscope (JEOL USA, Inc.).

### 2.6 Immunoblot analysis of SHED-MSC-EXOs by flow cytometry and western blotting

Purified exosomes were immersed in 100 μl of a solution containing 4 μm aldehyde/sulfate latex beads, and surface absorption was performed at room temperature. After the beads were washed in 10 mM PBS pH 7.4, these particles were treated with fluorescein-conjugated antibodies directed against exosomal membrane markers such as CD9, CD63, and CD81, or an appropriate isotype control. BD FACS Calibur flow cytometer (California, USA) was used to examine stained beads suspended in 100 µl FACS buffer (0.8% BSA in 10 mM PBS pH 7.4). The obtained data were analyzed using FlowJo software. All materials for the flow cytometry process (buffers, CD antibodies) were sourced from Biolegend Co. Western blotting focuses on CD9, CD63, and CD81 as exosome membrane markers. A combination of 25 μl of SHED-MSC-EXOs and 5X sample buffer (including 250mM Tris pH 6.8, 40% Glycerol, 20% β-mercaptoethanol, 0.01% Bromophenol blue, and 8% SDS) was subjected to heating at 95 °C for 5 minutes. The proteins were then separated through electrophoresis using a 12% SDS-PAGE gel. The gel was divided into two halves. One half was stained with Coomassie Brilliant Blue R250, while the other was transferred to a PVDF membrane (Bio-Rad Co., USA). The blotted membranes were incubated with primary antibodies targeting the EXO membrane markers CD9, CD63, and CD81. HRP-conjugated secondary antibodies (mouse anti-rabbit IgG-HRP) were used to bind with the blotted membranes before visualizing them using an enhanced chemiluminescence substrate. All antibodies were prepared by Santa Cruz Biotechnology (Inc. Santa Cruz, CA, USA). The SDS-PAGE electrophoresis, western blot, and R250 staining were performed based on Green & Sambrook (2012) manuals (19).

### 2.7 THP-1 macrophage polarization, staining, and flow cytometry

To create polarized (M0) macrophages, 5×10^5^ THP-1 monocytes were differentiated with PMA for 24 hours. The M0 macrophages were then further polarized into an inflammatory M1 state by culturing them in complete media containing 20 ng/mL LPS and 20 ng/mL IFN-γ for an additional 24 hours. Cells were detached with 5 mM EDTA at 4°C for 15 min. Cells were pretreated with Fc Block (BD Biosciences) and incubated for 30 min at 4°C with fluorochrome-conjugated primary antibodies targeting the M0 and M1 macrophage-specific markers CD14, CD68, CD80, and CD86. FACS buffer was used to wash cells twice while they were suspended in 0.5% PFA. Until an LSRFortessa flow cytometer (BD Biosciences) was used, cells were maintained in the dark at 4°C.

### 2.8 Labeling of SHED-MSC-EXOs and uptake by resulting macrophage cells

The staining process of SHED-MSC-EXOs using Calcein-AM (Sigma-Aldrich Co. USA) is a reliable method to determine their integrity. Calcein AM dye (1μl of 2 μM) was added to an equivalent number of EXOs based on 12 μg of exosomal protein and left to incubate in the dark at room temperature for 30 minutes. Intact EXOs will retain the green fluorescence emitted by Calcein that has penetrated their interior and been cleaved by internal esterases. After centrifugation at 3000 rpm for 40 minutes, the Calcein AM-labeled EXOs were isolated, while any non-penetrating Calcein AM was removed along with the supernatant. M0/M1 macrophages derived from THP-1 monocyte cells at a concentration of 2.5×10^5^/ml medium were incubated with labeled EXO for 6 h at 37°C. Using inverted fluorescence microscopy (Olympus, Japan), treated macrophage cells were observed.

### 2.9 Treatment of THP-1-derived macrophage cells

Cell culture experiments confirmed the activity of SHED-MSC-EXOs. We then added an equivalent number of EXOs based on 12 μg/ml exosomal proteins per well containing THP-1 monocyte-derived M0/M1 macrophages for 48 h. As a point of comparison, a separate group of cells was stimulated with 10 μg/mL of Dexamethasone (Farmingdale, NY, USA). This Dex. concentration was based on its similarity to an *in vivo* dose of 1 mg/kg body weight, as stated in the literature protocol. The study comprised two groups, M0 and M1, which were treated with isolated EXOs (Test), dexamethasone (positive control), or untreated (negative control). Each examination was repeated three times.

### 2.10 Collection of the supernatant and macrophage cells

After a 48-hour incubation period, the supernatant resulting from the treatment of macrophages with SHED-MSC-EXOs and dexamethasone was harvested by centrifugation at 3000 rpm for 10 minutes. It was then stored at -20°C for subsequent analysis, which included quantifying cytokines and determining oxidative stress index levels. Following the supernatant stored, the M0 and M1 macrophage cells adhering to the bottom of the culture plate were washed twice with ice-cold 0.1 M PBS pH 7.4. Subsequently, the macrophage cells were detached from the plates using ice-cold 10 mM EDTA pH 8 for 5 minutes. The separated macrophage cells were centrifuged at 3000 rpm for 3 minutes and stored to measure surface markers by flow cytometry.

### 2.11 Flow cytometry

The markers CD80 or CD86 (Classical M1-specific markers) and CD206 (M2-specific markers) were analyzed to determine the polarization and characteristics of macrophages. Conjugated and non-conjugated antibodies with fluorescence were used to investigate the cell surface markers. Non-specific fluorescence was adjusted by using appropriate isotype controls. The intensities of FITC-conjugated anti-human CD80, PE-conjugated anti-human CD86, and PE-conjugated anti-human CD206 antibodies were measured in EXO treated and non-treated macrophages. The efficiency of M2 macrophage polarization was determined by calculating the CD206/CD80,86 ratios for each differentiation. Samples were analyzed using a BD Accur flow cytometer (BD PharMingen, San Diego, CA) and BD Accur C6 Flow analysis software.

### 2.12 RNA extraction and real-time PCR

To extract total RNA from detached macrophage cells using Biozol (Bioer, China), the manufacturer’s instructions were followed. One microgram of total RNA with random hexamer primers (Bioron, Germany) was reverse-transcribed to generate cDNA and treated with DNaseI (CinnaGen, Iran). Real-time PCR was performed using Bioron SYBR green master mix (Bioron, Germany) with a Bioer Real-time PCR detection system (Bioer Technology, Hangzhou High Tech, China). *Glyceraldehyde 3-phosphate dehydrogenase* (*GAPDH*), an appropriate internal reference, was used to normalize gene expression. The mRNA expression levels of *Arg-1* (anti-inflammatory index and M2 marker) and *IL-6R* (pro-inflammatory cytokine and M1 marker) were measured to explore the effective M2 phenotype. Gene-specific primers that were designed and analyzed to span exon-exon junctions are listed in **Table 1**. All experiments were performed in triplicates and the mRNA expression level for each combination using the 2^ − ΔΔCt^ method.

**Table 1:** Gene-specific primers for RT-PCR.

Primer (accession)	Sequence (5’ > 3’)	Tm (°C)	Amplicon size
*GAPDH* (>NM_002046.7)	F: 5’ ACTTTGGTATCGTGGAAGGAC R: CAGTAGAGGCAGGGATGATG 3’	57	135
*Arg-1* (>NM_001244438.2)	F: 5’ GGTGGCAGAAGTCAAGAAGAAC R: GTGGTTGTCAGTGGAGTGTTG 3’	59	159
*IL-6R* (NM_001382774.1)	F: 5’ TCACTGTGTCATCCACGACG R: CTGGATTCTGTCCAAGGCGT 3’	59	132

### 2.13 Analysis of EXO-treated resulting macrophage supernatant

Pro- and anti-inflammatory cytokines levels were measured in culture supernatants of treated and untreated cells. To do this, specific cytokine ELISA kits (ZellBio GmbH Co. Germany) were used, following the instructions provided by the manufacturer. The optical density of each sample at a wavelength of 450 nm was determined using a Biotek ELISA reader model ELX800 (Biotek, USA). Each experiment for every sample was performed in triplicate to ensure accurate results. The data obtained were expressed as picograms of cytokines per ml.

To determine the levels of NO and MDA, which act as indicators of oxidative stress, the KPG kit (Karmania Pars Gene Co. Iran) was used. inducible nitric oxide synthase (iNOS) uses L-arginine, oxygen, and NADPH as substrates to produce NO, which is involved in many physiological processes, including immunity and inflammation. NO is very unstable and oxidized to nitrite and then to nitrate, which can be determined from cell culture or serum supernatant by the Griess colorimetric method. Lipid peroxidation is a consequence of cellular damage, which is known as a marker for assessing oxidative stress. Lipid peroxidation was performed by measuring the MDA product using the thiobarbituric acid or TBA method. In this method, malondialdehyde reacts with TBA reagent at high temperature (90-100°C) and this reaction produces two moles of H_2_O and one mole of MDA-TBA, and this product has absorption at the wavelength 535 nm. TAC levels and CAT and SOD activities as antioxidant indices were determined using a KPG kit (Karmania Pars Gene Co. Iran). TAC was measured using the FRAP method, which is based on the reducing ability of divalent iron through a single electron transfer mechanism. CAT activity was determined by quantifying the consumption of H_2_O_2_ per minute. A unit (U) of CAT enzyme is defined as 1 μmol H_2_O_2_ consumed per minute, and the specific activity was calculated by dividing U/mg of CAT proteinSOD activity was assessed spectrophotometrically by measuring the inhibition of pyrogallol autoxidation at 420 nm, which indicated the presence of O^2-^ as a SOD substrate.

### 2.14 Statistical analysis

Study results were presented using mean ± standard deviation (SD). Statistical analysis was performed using Prism 8 (GraphPad Software, San Diego, CA, USA) with an unpaired two-tailed T-test and one-way ANOVA. A p value of less than 0.05 was considered statistically significant.

## 3 Result

### 3.1 Quantification of isolated exosomes

Exosomes were quantified via a nanodrop spectrophotometer and a micro-Bradford assay to measure the total protein content. Both methods confirmed the presence of exosomes by indirectly evaluating the protein content of the sample. The findings indicated an average yield of 0.6 mg/ml.

### 3.2 Structure of SHED-MSC-EXOs at the nanoscale level

**Figure 2:**
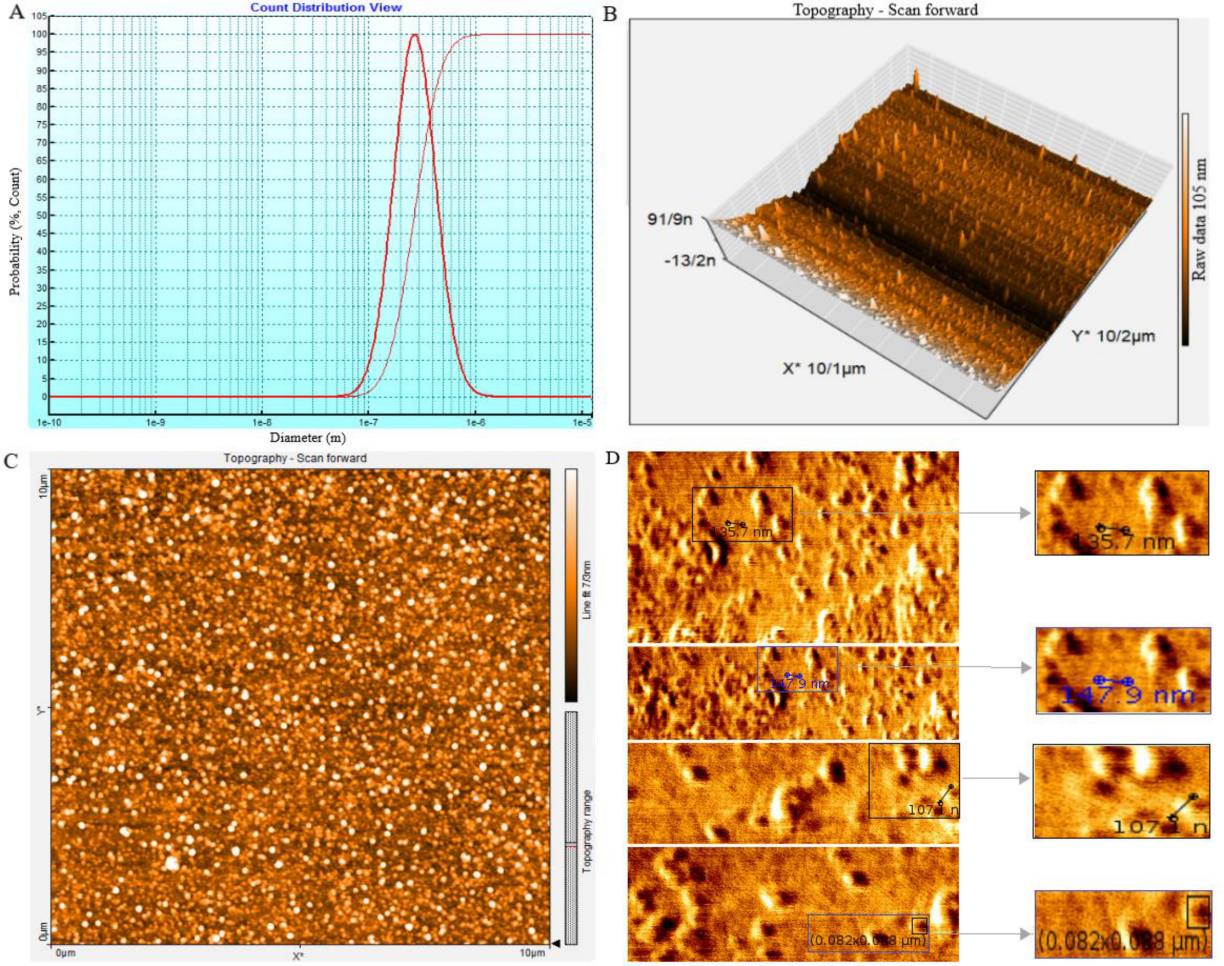
The SHED-MSC-EXOs ultrastructure analysis using DLS and AFM. **(A)** The average size of nanoparticles was determined by performing dynamic light scattering on SHED-MSC-EXOs. The results showed that the SHED-MSC secreted vesicles were approximately 10-100 nanometers in diameter. **(B, C)** The topography of SHED-MSC-EXOs was observed using AFM in tapping mode. When the EXOs were precipitated onto a mica surface, they exhibited distinct, uniform, regular, and relatively rounded structures in 2D and 3D images. **(D)** High-resolution images of SHED-MSC-EXOs acquired by AFM.

The size, morphology, and uniformity of particles derived from SHED-MSCs were assessed through DLS and AFM analysis. The DLS results showed that the size of the vesicles is around 100 nm (**Figure 2A**), which aligns with the AFM findings. Tapping mode, AM-AFM, and PM-AFM imaging methods were utilized to investigate sub-vesicular components’ three-dimensional arrangement and structure. Each produces images with specific imaging forces and feedback parameters (**Figures 2B–D**).

**Figure 3:**
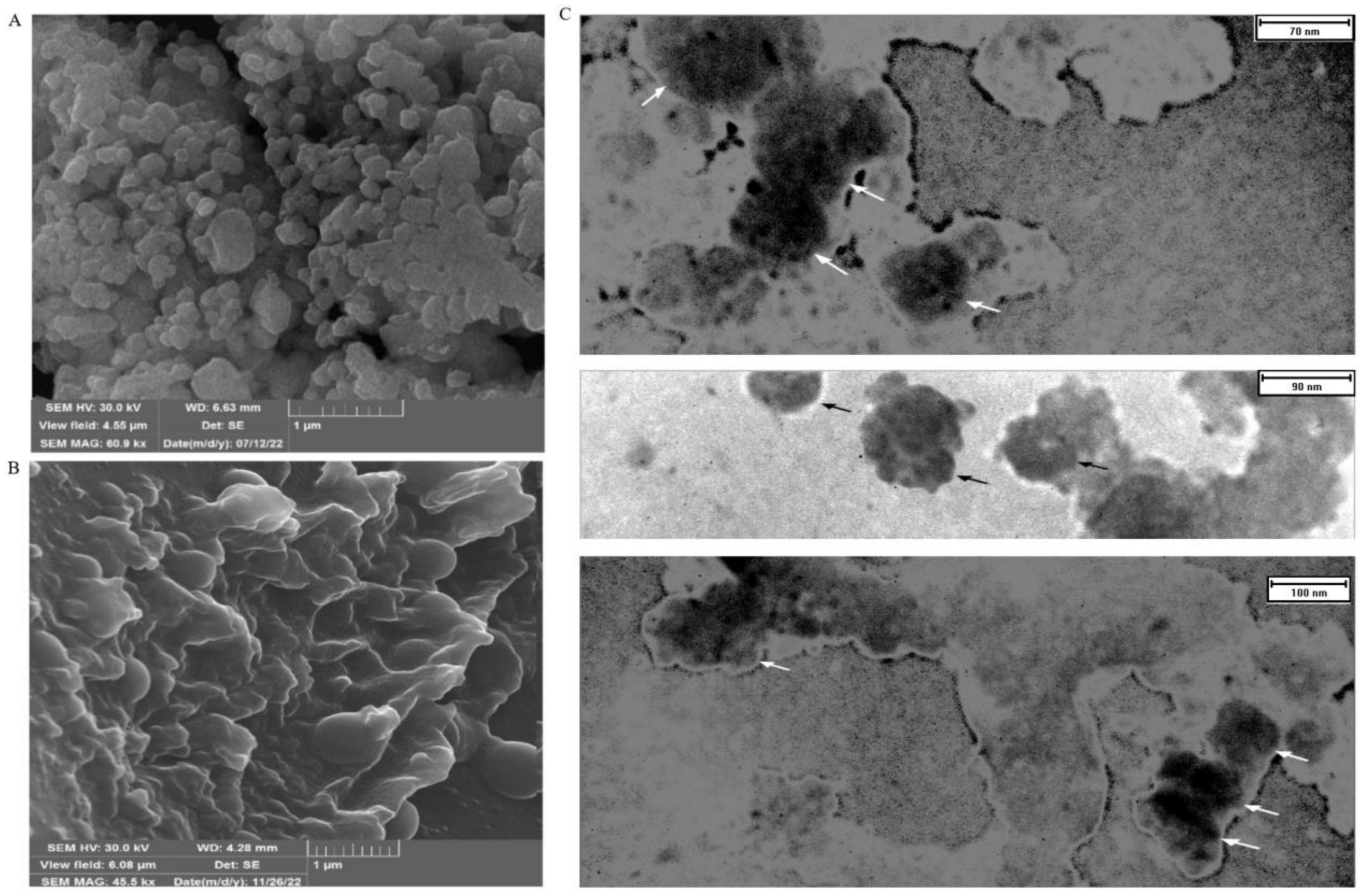
FESEM and TEM make it possible to observe the shape and size of SHED-MSC-EXOs. **(A)** EXOs are shown after freeze-drying without being diluted in water. **(B)** display the EXO after being resuspended and diluted in water following freeze-drying. The FESEM images show a visual depiction of EXO accumulation and individual vesicles, which appear as circular bulging structures without a central depression. These structures also exhibit clear inter-vesicular connections. **(C)** indicates EXOs imaged through TEM with size heterogeneity, fairly rounded, and characteristic cup-shaped morphology.

The use of FESEM has made it possible to conduct a detailed examination of the structural features of EXOs. This allows for a thorough comparison with high-resolution parallel imaging (**Figures 3A, B**). TEM analysis of multiple EXOs has shown round vesicles without any detectable internal structure. These imaging techniques also yield valuable information about the physiochemical properties of EXOs, providing further insights (**Figure 3C**).

### 3.3 Analysis of EXO surface proteins

**Figure 4:**
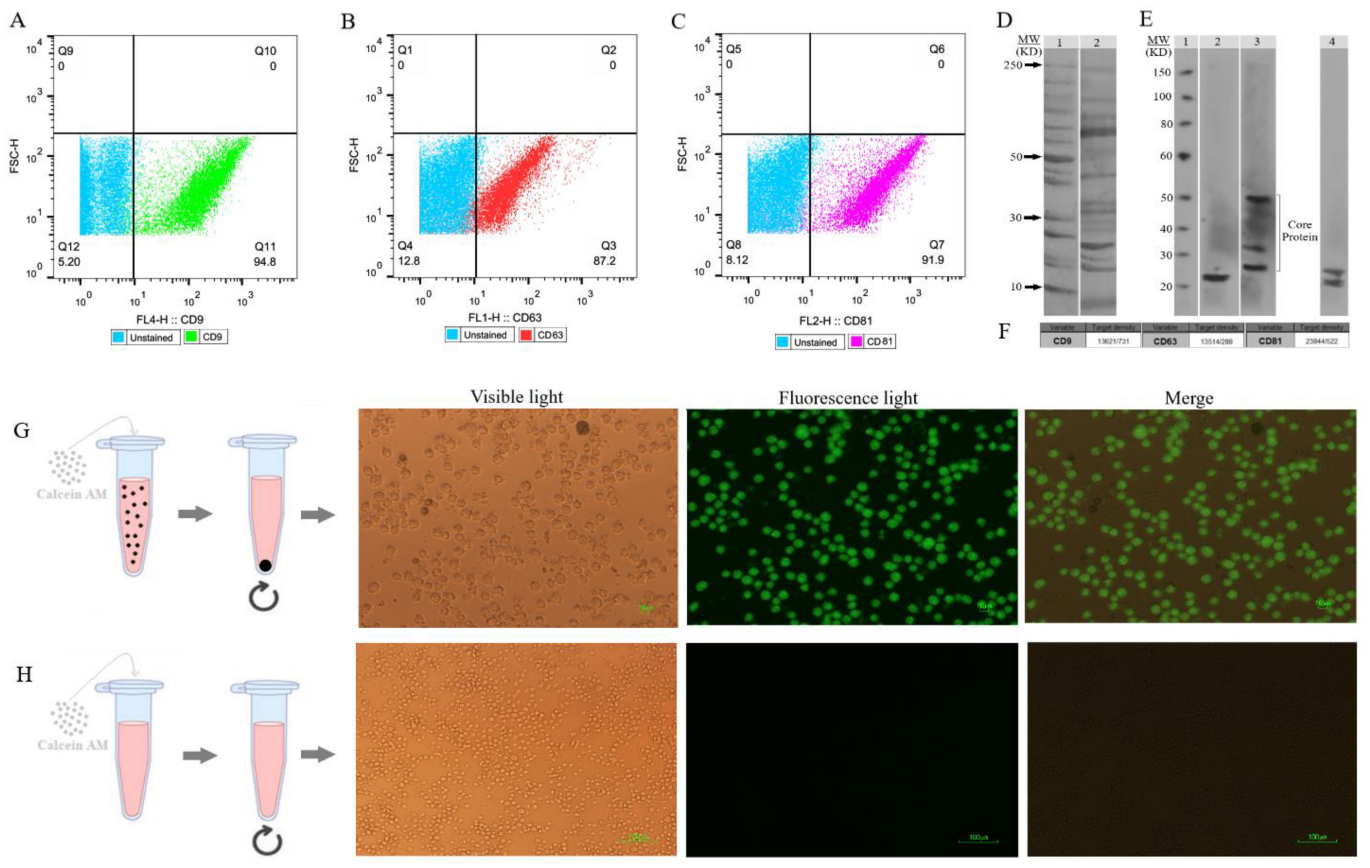
SHED-MSC-EXOs surface markers profiling and internalization. **(A–C)** Appropriate gating in flow cytometry was used to identify the CD9, CD63, and CD81 marker intensity. **(D)** Analyzing the intrinsic proteins of the SHED-MSC-EXOs using SDS-PAGE. Molecular weight markers (lane 1), EXO pellet (lane 2). **(E)** A Western blot was performed to detect the expression of CD9 (24 kDa in lane 2), CD63 (26, 30 and 50 kDa in lane 3), and CD81 (22 and 26 kDa in lane 4) in EXOs. The presence of CD63 in lane 3 showed a smear pattern within the molecular weight range of 30-60 kDa, which can be attributed to its extensive glycosylation. Also, Non-specific bindings or aggregate formations of CD81 with other tetraspanins are represented by higher molecular weight bands in CD81. **(F)** The band’s signal intensity. **(G)** During EXO internalization, centrifugation is used to separate EXOs that contain calcein AM. When Calcein AM-labeled EXOs are taken up by the resulting macrophage cells, detectable green fluorescence is observed by inverted fluorescence microscopy. **(H)** Unlike labeled EXOs, non-penetrating Calcein AM is not separated during centrifugation and is removed with the supernatant. This group can serve as a negative control.

The flow cytometry technique assessed the presence of CD9, CD63, and CD81 protein markers on the EXOs’ surface (**Figures 4A–C**). These markers, CD9, CD63, and CD81, are commonly found on EXOs, but their quantities may vary among different subpopulations. Magnetic microspheres coated with anti-CD9, -CD63, and -CD81 antibodies were incubated in two groups with and without SHED-MSC-EXOs. After that, these exosomes were stained with either anti-CD63, anti-CD9, or anti-CD81 antibodies for exosome-specific marker detection. The findings indicated that all three markers were present on the surface of isolated particles. The highest percentage, 94.8%, was achieved with magnetic microspheres that were coated with anti-CD9 antibodies.

### 3.4 Analysis of SDS PAGE and western blotting

The proteins found in EXOs obtained from SHED were separated using 1D SDS-PAGE (**Figure 4D**). Furthermore, the Western blot analysis revealed the presence of EXO-associated proteins CD9, CD63, and CD81 in the MSC-derived EVs (**Figure 4E**). **Figure 4F** illustrates the quantification of the signal emitted by the protein band(s). The intensity of the signal from the band is directly related to the amount of the target protein present.

### 3.5 SHED-MSC-EXOs labeling and uptake

To evaluate the absorption of EXOs, we tagged them with Calcein AM and added them to the cell culture medium of M0 and M1 macrophages. After 6 and 24 hours, we noted a noteworthy rise in the percentage of macrophage cells displaying intense green fluorescence when examined using inverted fluorescence microscopy (**Figures 4G, H**). The fluorescence strength amplified as the duration of incubation advanced.

### 3.6 THP-1 derived M0/M1 macrophage cells

**Figure 5:**
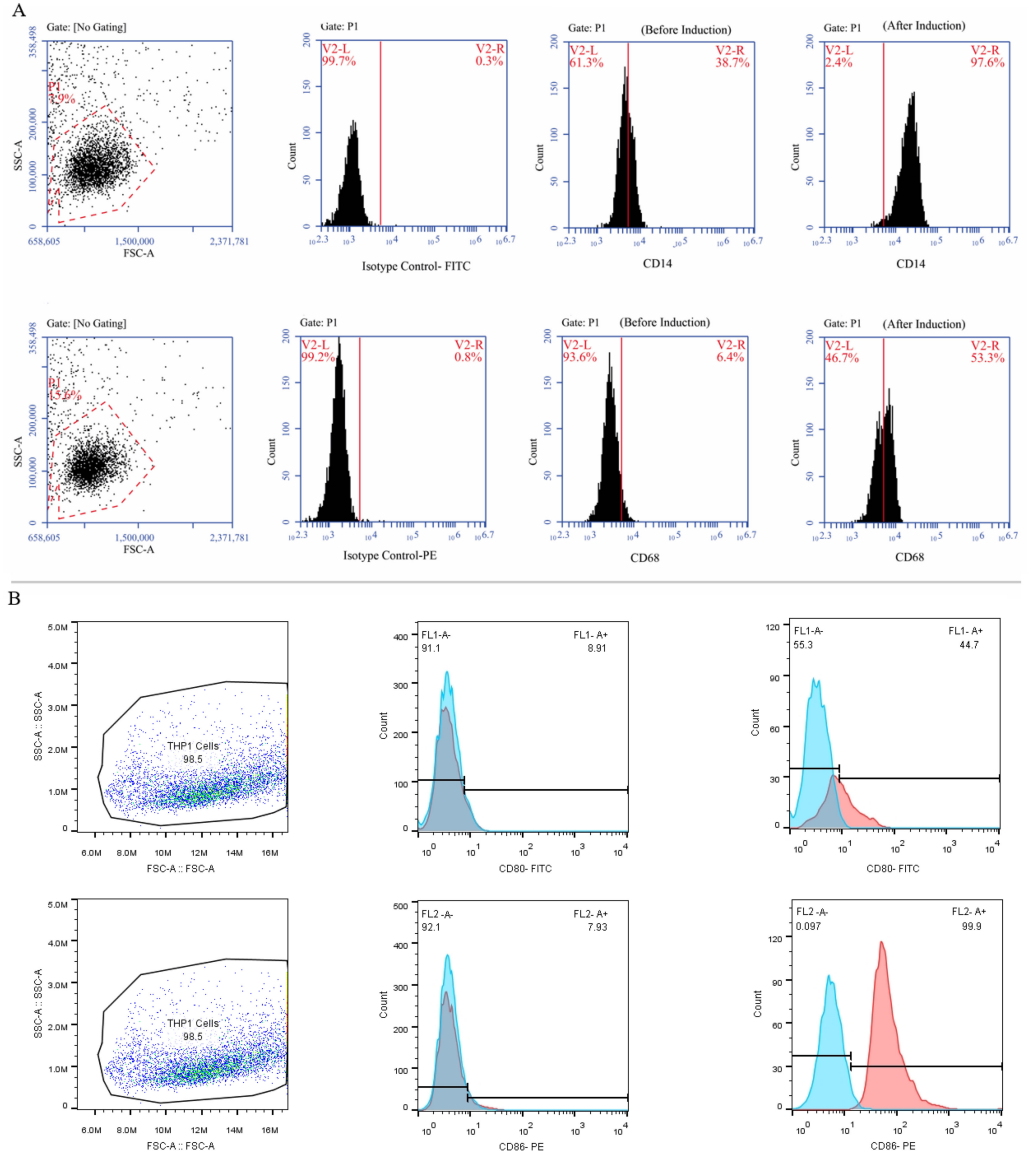
The process of differentiating THP-1 monocytes into macrophage cells. **(A)** The THP-1 cells were treated with PMA in the DMEM-F12 medium for 24 hours and the morphological changes were carefully observed using an inverted microscope at a 100x magnification. Subsequently, the cells were marked, and their expression of CD14 and CD68 (as M0-specific markers) was confirmed using specific antibodies. **(B)** Treatment of M0 cells with 20 ng/ml IFN-γ and 100 nmol LPS polarized them into M1 macrophages and an inverted microscope was utilized to examine morphological changes at a 100x magnification. Additionally, the cells were marked, and their expression of CD80 and CD86 was validated using flow cytometry.

Following a 24-hour incubation with 20 ng of PMA, human THP-1 monocytes differentiated into M0 macrophage cells. The M0 cells exhibited adhesion and increased expression of M0-specific markers, including CD68 and CD14, as indicated by Immunofluorescence staining (**Figure 5A**). By the established literature protocol for M1 differentiation, the M0 cells were exposed to 20 ng/ml IFN-γ and 100 ng/ml LPS in the DMEM-F12 medium for 24 hours. Subsequently, Immunofluorescence staining was performed to assess the resulting elevation in the expression of M1-specific markers, CD80 and CD86, thereby confirming the successful differentiation into M1 macrophages (**Figure 5B**).

### 3.7 Morphology of exosome-treated macrophage cells

**Figure 6:**
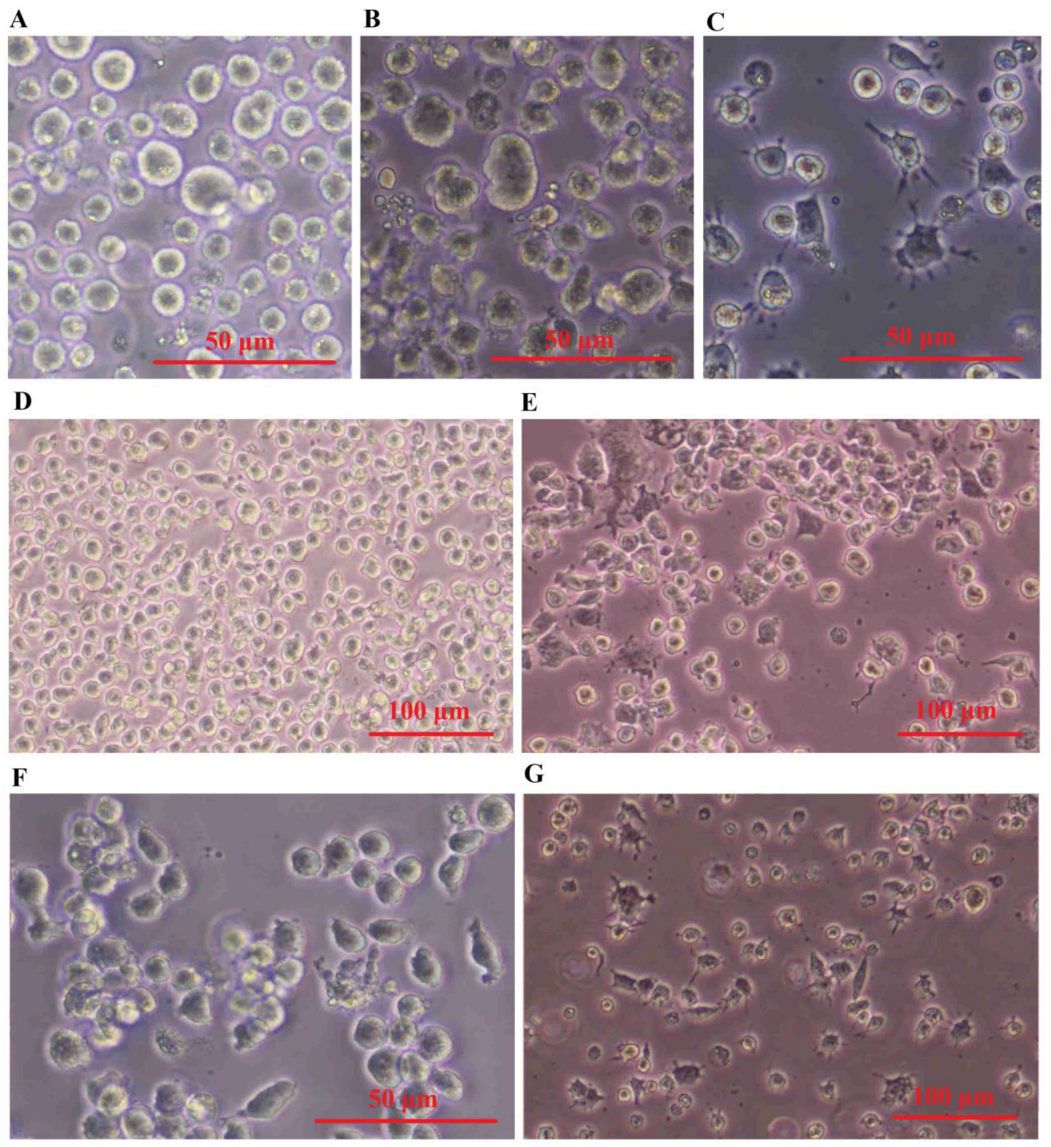
SHED-MSC-EXOs induced the M2 phenotype in both M0 and M1 macrophages. **(A)** Cell morphology of THP-1. **(B)** PMA-(M0)-induced THP-1 cells. **(C)** LPS & INF-Y-(M1)- induced M0 cells. **(D)** M0 and **(E)** M1 macrophage cell treated by Dex. **(F)** M0 and **(G)** M1 macrophage cell treated by SHED-MSC-EXOs.

THP-1 monocytes that have undergone differentiation have been extensively utilized as a laboratory model for human macrophages. To obtain different populations of macrophages - M0 phenotype (non-activated) and M1 phenotype (classically or pro-inflammatory) serial dose and time optimization experiments were performed on THP-1 cells. The results presented in **Figure 6** demonstrate that the monocyte-like THP-1 cells exhibit a distinct morphology, appearing as large, round single-cell clusters when suspended in culture medium (**Figure 6A**). In contrast, M0 cells adhere to surfaces and display a small, round shape with no cytoplasmic extensions (**Figure 6B**). M1 cells are identified by their round shape as they adhere to surfaces and have delicate protrusions made of cytoplasm without organelles, enclosed by regular membranes and coatings. These cells exhibit branching or fried egg-like structures in terms of morphology (**Figure 6C**). Conversely, M2 cells appear as adherent elongated “spindle-like” shapes that can be observed in **Figures 6D–G**.

### 3.8 Exosome-treated M0 macrophage cells increase M2-specific markers

**Figure 7:**
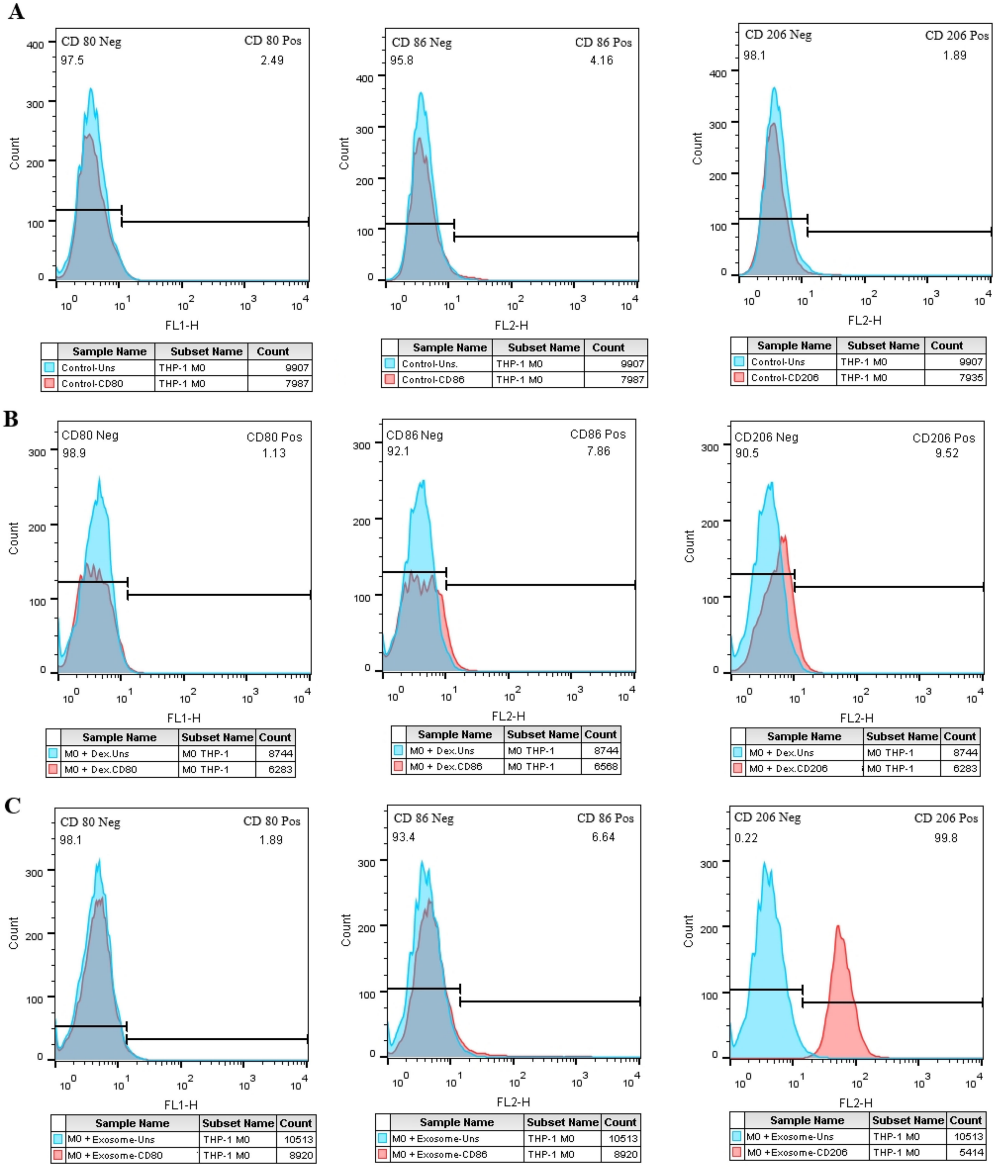
The effect of SHED-MSC-EXOs on M1/M2-specific markers in M0 macrophage cells. **(A)** Macrophage cells (M0) that were not treated. **(B)** M0 macrophage cells treated with Dex. **(C)** The percentage of cells positive for CD206 increases in the test group compared to the negative and positive control groups in exosome-treated M0 macrophage cells.

The expression of M1-specific markers (CD80 and CD86) and a marker specific for M2 macrophages, CD206, were measured by flow cytometry. Compared to the negative control group (**Figure 7A**) the M0 group treated with EXOs showed a significant rise in the expression of CD206 (**Figure 7C**), similar to the M0 group treated with dexamethasone as the positive control (**Figure 7B**). There were no noticeable changes in the expression of the M1 markers.

### 3.9 Exosomes adjust M2 and M1 specific markers in THP-1 derived M1 macrophages

**Figure 8:**
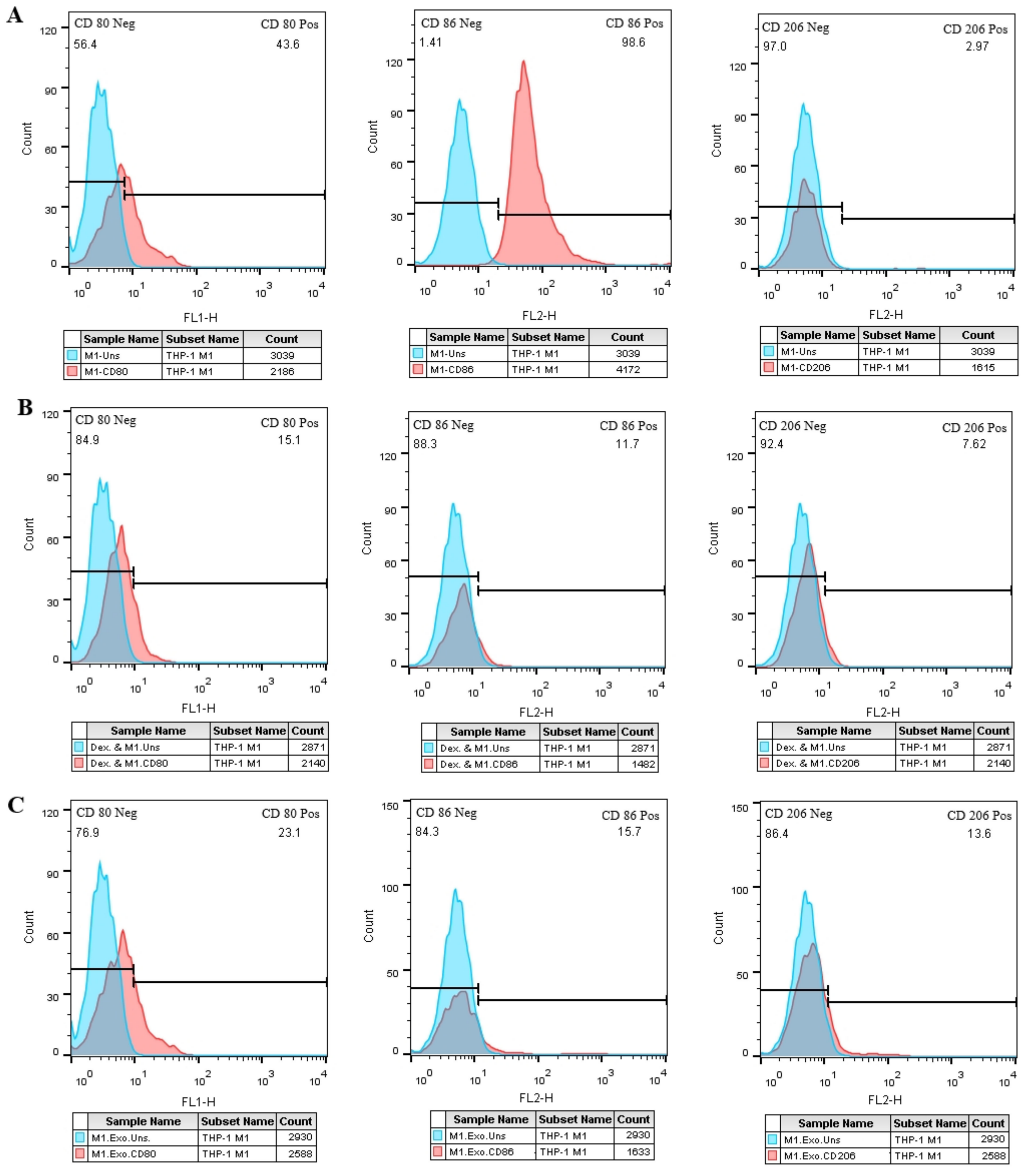
SHED-MSC-EXOs impact M1 and M2 specific markers in M1 macrophage cells. **(A)** M1 macrophage cells without treatment. **(B)** M1 macrophage cells treated with Dex. **(C)** M1 macrophage cells treated with EXOs. Compared to the control groups, M1 macrophages exhibit higher CD206-positive cells after EXO treatment. Conversely, the rate of CD86-positive cells decreases in M1 macrophages following exposure to EXOs compared to the negative control. These results emphasize the potential role of EXOs in modulating the phenotype and function of macrophages.

In comparison to the negative control group (**Figure 8A**), the M1 cell group that was treated with EXOs exhibited an increase in CD206 expression and a significant reduction in CD86 expression (**Figure 8C**), which was similar to the M1 cells that were treated with dexamethasone. However, unlike the positive control group, there were no significant changes in CD80 expression observed in the test group (**Figures 8B, C**).

### 3.10 SHED-MSC-EXOs reduced the pro-inflammatory cytokines and pro-oxidant indexes

**Figure 9:**
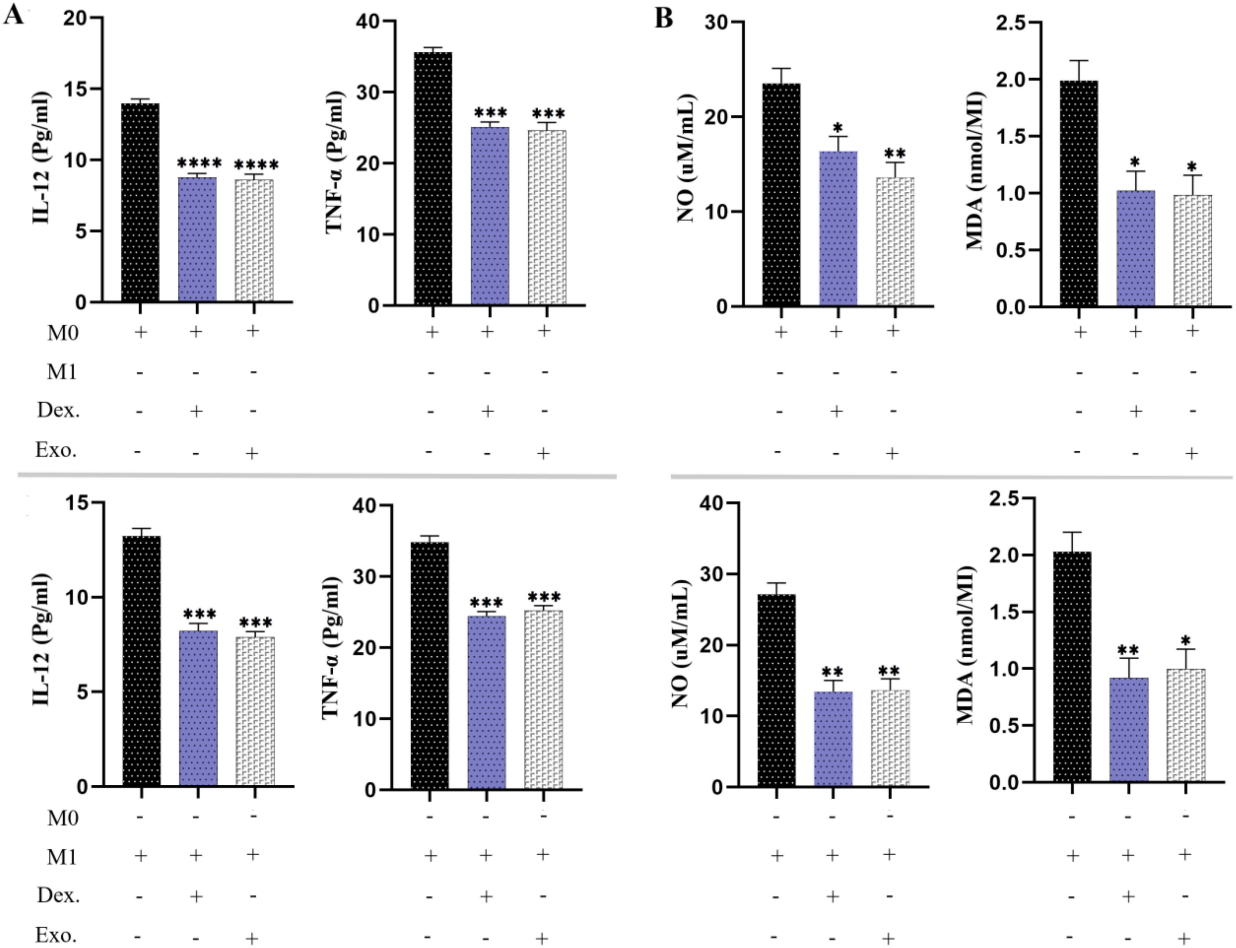
SHED-MSC-EXOs can reduce inflammatory stimuli in resulting macrophage cells. **(A)** The ELISA test was conducted on M0 and M1 macrophages to measure the pro-inflammatory cytokine levels of TNF-α and IL-12. **(B)** The Griess assay and TBARS method were utilized to evaluate the production of NO and MDA by macrophages. The mean SD of three independent experiments is indicated by each bar. ^∗^p < 0.05; ^∗∗^p < 0.01; ^∗∗∗^p <0.001; ^∗∗∗∗^p < 0.0001; ns, not significant.

The effects of SHED-MSC-EXOs on the inflammatory properties of M0 and M1 macrophages were assessed by analyzing the levels of IL-12, TNF-α, NO, and MDA in a culture medium treated with 12 μg/ml of isolated EXOs for 48 hours. As depicted in **Figure 9**, the results of the ELISA test revealed that the presence of SHED-MSC-EXOs, like dexamethasone, led to a reduction in the concentrations of IL-12 and TNF-α. Furthermore, the Griess assay and TBARS method demonstrated that isolated EXOs decreased the levels of nitrite and MDA.

### 3.11 SHED-MSC-EXOs has boosted the anti-inflammatory cytokines and anti-oxidant indicators

**Figure 10:**
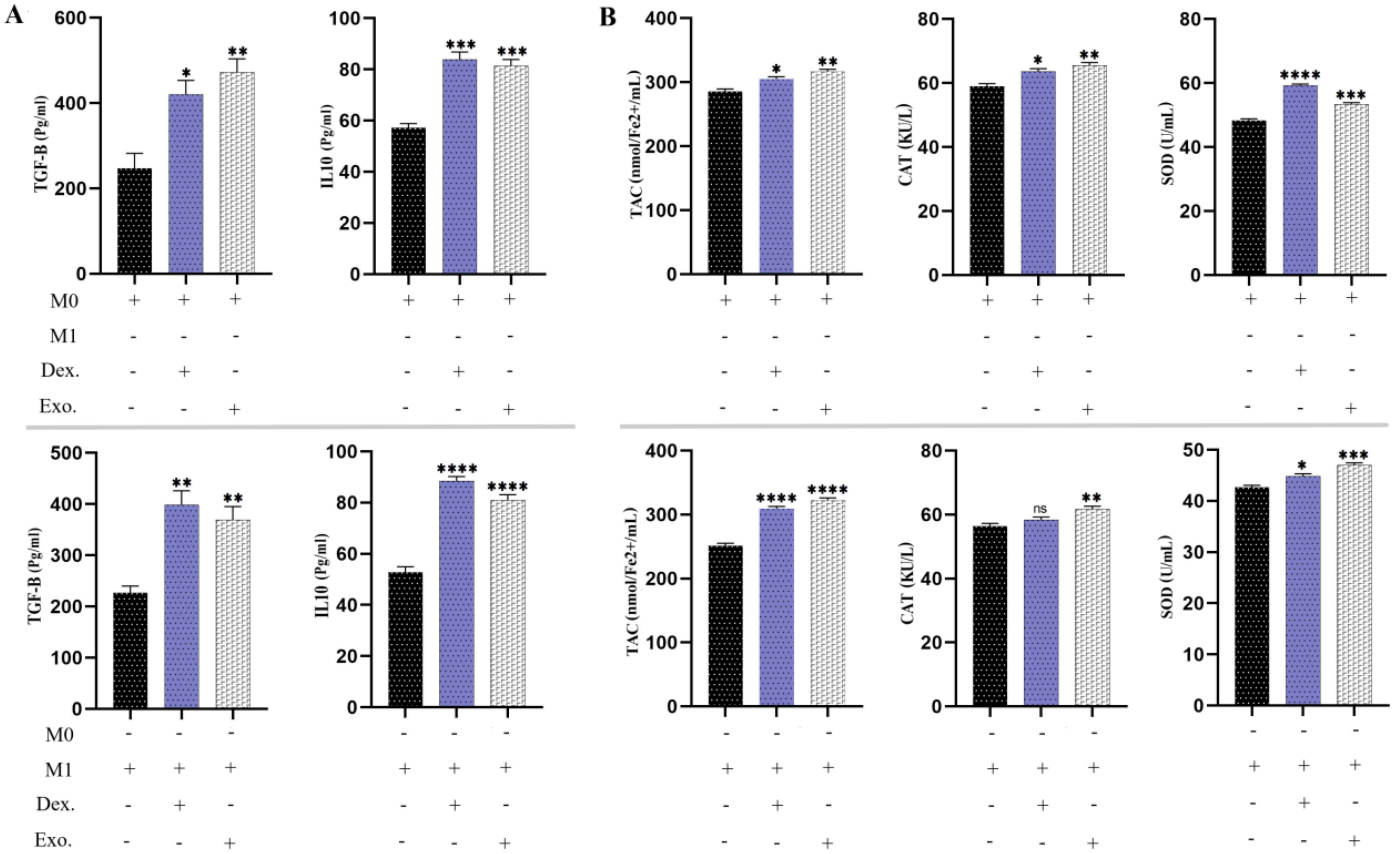
SHED-MSC-EXOs indicate an increase in anti-inflammatory stimuli. **(A)** The levels of anti-inflammatory cytokines TGF-β and IL-10 were measured in M0 and M1 macrophages before and after treatment using an ELISA assay. **(B)** The FRAP technique, CAT activity test, and SOD activity test were conducted on M0 and M1 macrophage cells treated with EXOs, as well as control groups. The objective was to evaluate their ability to reduce the ferric-tripyridyltriazine (Fe^3+^-TPTZ) complex to ferrous tripyridyltriazine (Fe^2+^-TPTZ) through the anti-oxidants present in the sample at a low pH, decomposition of hydrogen peroxide (H_2_O_2_) and pyrogallol. The mean SD of three independent experiments is indicated by each bar. ^∗^p < 0.05; ^∗∗^p < 0.01; ^∗∗∗^p <0.001; ^∗∗∗∗^p < 0.0001; ns, not significant.

As we have demonstrated, the utilization of SHED-MSC-EXOs could boost the anti-inflammatory response of M0 and M1 macrophages. As a point of reference, we utilized 10 μg/mL dexamethasone in a separate group. According to **Figure 10**, the findings from the ELISA examination revealed that cells treated with EXOs displayed comparable results to those treated with dexamethasone. This indicates that isolated EXOs led to increased levels of TGF-β and IL-10 as anti-inflammatory indicators. Analysis of the culture medium also showed increased levels of TAC, CAT, and SOD as antioxidant indices.

### 3.12 Transcript expression of *Arg-1* and *IL-6R* mRNA

**Figure 11:**
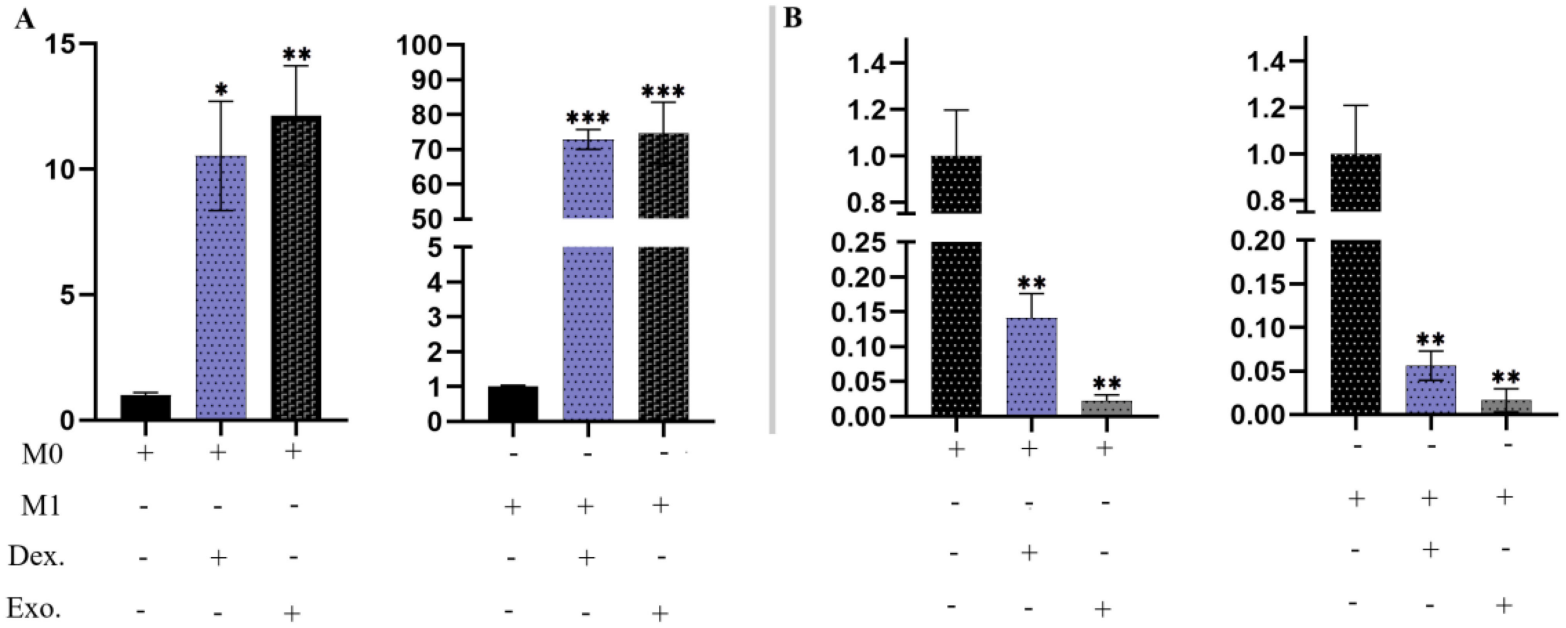
SHED-MSC-EXOs regulated the mRNA transcripts of *Arg-1* and *IL-6R.* **(A)** Treatment with isolated EXOs led to an increase in *Arg-1* expression in M0 and M1 Macrophage cells. **(B)** The resulting macrophages experience a downregulation of *IL-6R* due to SHED-MSC-EXOs treatment. All experiments were repeated in triplicates. Statistical significance was determined at p <.05. ∗p < 0.05; ∗∗p < 0.01; ∗∗∗p <0.001; ns, not significant.

The expression of *Arg-1* mRNA is a valuable indicator in anti-inflammatory macrophages, aiding in the investigation of damage repair. In **Figure 11A**, it is evident that macrophages treated with isolated EXOs and dexamethasone displayed increased levels of *Arg-1* (p <.05). Furthermore, we assessed the expression of *IL-6R*, a significant proinflammatory macrophage (M1) marker. Macrophages treated with SHED-MSC-EXOs and dexamethasone exhibited lower levels of expression *IL-6R* compared to the control group (p <.05), as illustrated in **Figure 11B**.

## 4 Discussion

**Figure 12:**
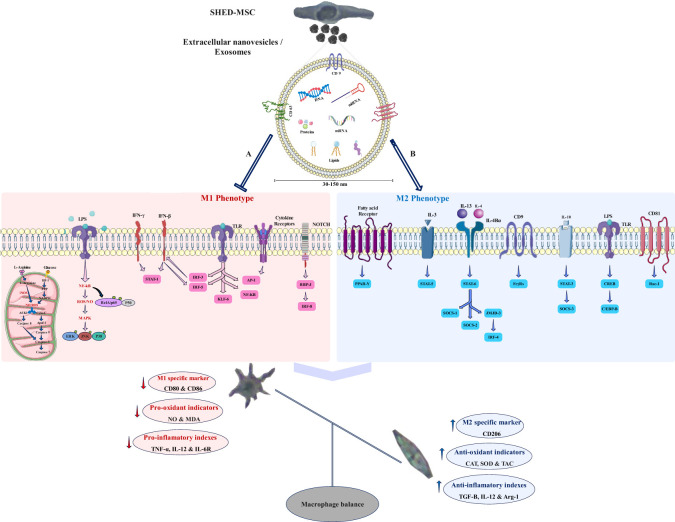
Potential signaling pathways that the exosome may regulate. SHED-MSC-EXOs likely modify the balance of macrophages and immunoregulatory macromolecules in M0/M1 polarized macrophages by inhibiting signaling pathway A and enhancing signaling pathway B, leading to a shift towards immunosuppressive macromolecules and the M2 phenotype. **(A)** Mitochondrial oxidative stress: An increase in HIF-1 α expression results in heightened activity of the glycolytic and pentose phosphate pathways, leading to greater production of NADPH oxidase and Inducible nitric oxide synthase enzymes, which subsequently generate hydrogen peroxide and nitric oxide in these cells. The levels of reactive oxygen species and nitric oxide can influence cell survival differently, depending on their concentration. They have the potential to activate the NF-κB pathway and disrupt cellular membrane homeostasis, which diminishes antioxidant defenses. This disruption may trigger the intrinsic mitochondrial apoptosis pathway through the activation of cytochrome c and the cleavage of caspase 3 and caspase 7. Additionally, it can initiate the extrinsic apoptosis pathway by activating ASK1 and JNK; Oxidative stress induced by LPS: Within the TLR/Myd-88/NF-κB signaling pathway, IκB undergoes degradation, and RelA (p65)/p50 gets phosphorylated, enabling their entry into the nucleus as a complex. For the activation of specific target genes regulated by NF-κB, the RelA (p65)/p50 subunit must engage with CBP/p300. The pathways activated by LPS-TLR and ROS-NFκB disrupt antioxidant defenses, leading to heightened release of pro-inflammatory cytokines; M1 macrophage polarization occurs when there is an elevation in the activation of NF-κB and STAT1, along with the stimulation of Notch1 and NF-kappa B by LPS and toll-like receptors, as well as the activation of IRF5 in response to inflammation. This results in a predominance of M1 macrophages, which perform proinflammatory functions that can harm tissues. Furthermore, the JAK-STAT signaling pathway is significantly linked to the functional characteristics of macrophages, and IFN influences their activities via this pathway. **(B)** The increase in M2 macrophage polarization, which is associated with immune tolerance and tissue repair, is mainly driven by the activation of STAT3 and STAT6 via IL-4/13 and IL-10. PPARδ and PPARγ play roles in regulating various aspects of M2 macrophage activation and oxidative metabolism. KLF-4, which is activated by STAT6, enhances M2 macrophage functions by suppressing NF-κB/HIF-1α-dependent transcription. IL-4 triggers the activation of c-Myc and IRF-4, which manage the expression of genes related to M2 polarization and inhibit IRF5-mediated M1 polarization. IL-10 promotes M2 polarization by activating the p50 NF-κB homodimer, c-Maf, and STAT3. CD9 interacts with FcγRs, influencing phagocytosis signaling pathways and inflammatory responses, while also inhibiting M1 macrophage activation by LPS and TNF-α by preventing the localization of CD14 into lipid rafts. CD81 interacts with Rac1 to block the downstream activation of STAT1 through interferon-alpha/beta receptor signaling.

SHED-MSCs do not show MHC II expression and have no high immunogenicity, indicating that they could be appropriate for cell therapy (20–22). However, concerns regarding potential side effects associated with MSC-based treatments led to exploring alternative therapeutic options, such as the SHED-MSC-derived exosomes (23). EXOs, acting as crucial paracrine messengers, harbor diverse bioactive compounds including proteins, lipids, signaling molecules, miRNAs, and mRNAs (**Figure 12**). These exosomes can act as nanocarriers, transporting bioactive substances from their source cells to recipient cells, thereby impacting the functions of the recipient cells (13). EXOs can deliver their contents to target cells through signaling interactions involving ligand/receptor molecules on cell surfaces, or by merging with the cell membrane via mechanisms such as clathrin and lipid raft-mediated endocytosis, macro-pinocytosis, and phagocytosis (24). Many studies have demonstrated that MSC-derived EXOs have significant therapeutic benefits in wound healing across various tissues (9, 25, 26) and do not provoke noticeable immune responses in non-immune-compatible animals, as they lack MHC class I or II molecules (27, 28). However, additional research on exosomes from different types of stem cells is necessary to examine their immunoregulatory roles in therapeutic applications. Consequently, the research is designed to explore the immunomodulatory function of SHED-MSC-EXOs in regulating the balance of immunoregulatory macromolecules and signaling pathways in THP-1-derived M0/M1 macrophage cells (**Figure 1**).

As shown in **Figure 1**, FBS was gradually phased out to isolate EXOs. These nanoscale particles were obtained from cells cultured in a serum-free medium for 48 hours. The morphology and dimensions of the EXOs were determined using DLS, AFM, FESEM, and TEM. **Figures 2**, **3** illustrate that these particles exhibited rounded protrusions with no central depression, connections between vesicles, and a cup-shaped structure. The EXOs varied in size from 50 to 150 nm, with most having a diameter of around 88 nm. Flow cytometry and Western blotting were used to validate the presence of CD9, CD63, and CD81 markers in SHED-MSC-EXOs surface (**Figures 4A–E**). In the western blot analysis, CD63 displayed a smear pattern in the molecular weight range of 30-60 kDa, likely due to its significant glycosylation (**Figure 4E**, lane 3). Additionally, higher molecular weight bands in CD81 indicate non-specific binding or aggregation with other tetraspanins (**Figure 4E**, lane 4). Calcein AM staining revealed that the SHED-MSC-EXOs are intact vesicles, which is crucial for their effectiveness (**Figures 4G, H**). These findings confirm the successful isolation of EXOs from SHED-MSCs (**Figure 1A**). The investigation into the polarization of M0/M1 macrophages derived from THP-1 monocyte cells at a concentration of 2.5×10^5^/ml medium revealed increased expression of specific markers at the M0 (CD14, CD68) and M1 (CD80, CD86) that showed in **Figure 5**. The analysis of pro-inflammatory and pro-oxidant indicators such as CD80, CD86 (**Figures 7**, **8**), TNF-α, IL-12, NO, MDA (**Figure 9**), and *IL-6R* (**Figure 11B**) and anti-inflammatory and antioxidant markers including CD206 (**Figures 7**, **8**), TGF-β, IL-10, TAC, CAT, SOD (**Figure 10**), and *Arg-1* (**Figure 11A**) in the supernatant and the polarized THP-1 cells demonstrated exosome treatment reduced the level of immunostimulatory macromolecules and increasing the level of immunosuppressive macromolecules in M0/M1 macrophage cells.

In the analysis of the supernatant and cells within each group, it was discovered that these nano-vesicles, similar to the functionality of dexamethasone, can decrease pro-inflammatory and pro-oxidant markers levels and increase anti-inflammatory and antioxidant indicators within resulting M0 and M1 macrophage cells (**Figure 12**). Indeed, exosomes from SHED-MSCs can inhibit M1 polarization by decreasing the expression of CD86, a marker linked to M1 macrophages (**Figure 12A**). Also, these nanoparticles can promote the change of M0 and M1 macrophages into M2 macrophages by increasing the expression of M2-specific markers such as CD206 (**Figure 12B**). Previously, using a rat model of traumatic brain injury (TBI), the administration of SHED-MSC showed anti-inflammatory effects, which aligns with our findings (9). The mechanisms by which exosomes regulate the immune system are still not completely understood. Studies have shown that EXOs transport various molecules, including RNAs, proteins, and lipids, to targeted cells, and alter their behavior (29).

Certain proteins found in MSC exosomes have been shown to affect macrophage behavior. TGF-β encourages the M2 macrophage phenotype, which is associated with anti-inflammatory responses and tissue repair (30). When IL-10 binds to its receptor complex, it triggers the phosphorylation of JAK1 and the activation of STAT3, which in turn inhibits pro-inflammatory cytokines like TNFα, IL-1β, IL-12, and IFNγ. This anti-inflammatory cytokine also encourages macrophages to shift towards the M2 phenotype, enhancing their capacity to alleviate inflammation (17, 31). HGF has been shown to enhance macrophage survival and modify their activation state (32). VEGF can activate macrophages and promote angiogenesis, which is crucial for tissue repair and regeneration (33). As illustrated in **Figure 12**, CD9, a functional protein in exosomes, can inhibit LPS and TNF-α pathways in M1 macrophages by interacting with FcγRs and preventing CD14 from localizing to lipid rafts (34, 35). CD81, recognized as a specific exosomal marker, interacts with Rac1 to block the subsequent activation of STAT1 through the interferon-alpha/beta receptor signaling pathway (34).

Alongside functional proteins, eight miRNAs identified in SHED-MSC-EXOs, including hsa-miR-1260a, hsa-miR-4281, hsa-miR-4516, hsa-miR-4530, hsa-miR-5787, hsa-miR-6090, hsa-miR-6125, and hsa-miR-6510-5p, can target specific genes related to M1 macrophage polarization, mitochondrial oxidative stress, and LPS-induced oxidative stress pathways (17). Additionally, miR-21 (36), miR-126 (37), miR-223 (38), and miR-155 (39) are recognized for promoting macrophage polarization toward the M2 phenotype, which aids in resolving inflammation.

As shown in **Figure 12**, exosome contents in macrophage cells can suppress the TLR4/NF-κB pathway (40), stimulate the PI3K/AKT pathway (28), and elevate the levels of PPARγ (1) and adenosine triphosphate (41). These nanoparticles also influence various signaling pathways related to cell growth, including the Epidermal Growth Factor Receptor pathway, where proteins like nuclear factor erythroid 2-related factor 2 (Nrf2) and rapidly accelerated fibrosarcoma are involved (42, 43). Nrf2 is a key regulator of the antioxidant response, as it governs the expression of phase II detoxification and antioxidant enzymes. Genes controlled by Nrf2 can bolster the body’s antioxidant defenses and lower reactive oxygen species (ROS) production, which in turn can reduce inflammation (44, 45).

These insights are crucial for developing new therapeutic approaches aimed at diseases linked to macrophage function and enhance our understanding of how SHED-MSC-EXOs can be utilized in treating inflammatory conditions, autoimmune disorders, and regenerative medicine. The distinctive physical characteristics of exosomes, including their nanoscale size, lipid membrane, and ability to easily enter target cells, present a promising avenue for therapeutic applications. However, further research is necessary to investigate their potential and refine their application in clinical environments.

## 5 Conclusion

In recent years, scientists have directed their attention to investigating macrophage polarization to create treatment strategies that modulate the immune system for various diseases. The polarization of macrophages can be achieved by directly interacting with SHED-MSCs or indirectly through the extracellular substances known as the secretome. The primary components of the secretions from SHED-MSCs are soluble factors, proteins, and extracellular vesicles/MSC-EVs, particularly exosomes, which play a crucial role in tissue repair and regeneration due to their immunomodulatory effects.

Here, following isolation, characterization, and internalization of exosomes, macrophage-polarized THP-1 cells were exposed to 12 µg/ml of SHED-MSC exosomes and 10 µg/ml of dexamethasone. The *in vitro* results revealed that exosomes derived from newly cultured SHED-MSCs had a size ranging from 50 to 150 nm, with a diameter of approximately 88 nm, and a concentration of 0.6 mg/ml. The isolated exosomes displayed raised and rounded surfaces without central depression in the FE-SEM study, and inter-vesicular junctions and cup structure in the TEM analysis.

Additionally, the high expression levels of exosome markers CD9, CD63, and CD81 were confirmed through indirect immunofluorescence and western blot analysis. Also, Calcein AM staining revealed that the SHED-MSCs exosomes are indeed intact vesicles, which is crucial for their effectiveness. Overall, our study found that purified SHED-MSC-EXOs can inhibit M1 macrophages, promote the M2 macrophage phenotype, and enhance anti-inflammatory cytokines and antioxidant levels while reducing pro-inflammatory cytokines and pro-oxidants. The components found in EXOs likely play a role in suppressing the activity of STAT1, NF-κB, specific receptors such as TLR4, as well as enzymes like iNOS and NOX (NADPH oxidase). This inhibition leads to a decrease in the production of immunostimulative macromolecules. Besides, the expression of STAT6, STAT-3, KLF4, KLF2, *Arg-1*, and Fizz-1 is upregulated, increasing the immunosuppressive macromolecules.

Indeed, our study emphasizes the therapeutic potential of using stem cell-derived nanoparticles like SHED-MSC-EXOs to modulate immune cells such as macrophages. Probably, SHED-MSCs exosomes can minimize inflammatory responses and tissue damage associated with M1 macrophage-induced diseases. Low immunogenicity and strong regulation of the immune system, making exosomes a promising cell-free therapy for many diseases, especially in combination with chronic inflammation. However, further research is necessary to fully comprehend their mechanisms of action and evaluate their effectiveness in clinical settings.

## Data Availability

The original contributions presented in the study are included in the article/supplementary material. Further inquiries can be directed to the corresponding authors.
